# Detection of antibodies against the CB9 to ICB10 region of merozoite surface protein-1 of *Plasmodium vivax* among the inhabitants in epidemic areas

**DOI:** 10.1186/1475-2875-13-311

**Published:** 2014-08-12

**Authors:** Tong-Soo Kim, Youngjoo Sohn, Jung-Yeon Kim, Won-Ja Lee, Byoung-kuk Na, Yoon-Joong Kang, Hyeong-Woo Lee

**Affiliations:** Department of Parasitology, College of Medicine, Inha University, Incheon, 405-751 Republic of Korea; Department of Anatomy, College of Korean Medicine, Institute of Korean Medicine, Kyung Hee University, Hoegi-dongDongdaemun-gu, Seoul 130-701 Republic of Korea; Division of Malaria and Parasitic Diseases, National Institute of Health, Korea Centers for Disease Control and Prevention, Osong, 363-951 Republic of Korea; Department of Parasitology and Institute of Health Sciences, Gyeongsang National University School of Medicine, Jinju, 660-751 Korea; Department of Biomedical Science, Jungwon University, Goesan, Chungbuk 367-805 Republic of Korea; Department of Pathology, Immunology, and Laboratory Medicine, College of Medicine, University of Florida, Florida, FL 32610 USA

## Abstract

**Background:**

The purpose of this study was to examine the usefulness of the conserved block 9 (CB9) to interspecies conserved block (ICB10) region of *Plasmodium vivax* merozoite surface protein-1 (MSP-1 (ICB910)) as a serodiagnostic tool for understanding malaria transmission.

**Methods:**

Antibody titre in the blood samples collected from the inhabitants of Gimpo city, Paju city and Yeoncheon county of Gyeonggi Province, as well as Cheorwon county of Gangwon Province, South Korea were determined by enzyme-linked immunosorbent assay (ELISA). Microscopic examination was performed to identify malarial parasites.

**Results:**

MSP-1(ICB910) is encoded by a 1,212-bp sequence, which produced a recombinant protein with a molecular weight of approximately 46 kDa. Antibody titres in 1,774 blood samples were determined with the help of ELISA using purified recombinant MSP-1(ICB910). The overall ELISA-positive rate was 8.08% (n = 146). The annual parasite incidences (APIs) in the regions where the blood sampling was carried out gradually decreased from 2004 to 2005 (1.09 and 0.80, respectively). Yeoncheon county had the highest ELISA-positive rate (10.20%, 46/451). Yeoncheon county also had the highest API both in 2004 and 2005, followed by Cheorwon county, Paju city and Gimpo city.

**Conclusions:**

The MSP-1 (ICB910)-ELISA-positive rates were closely related to API in the geographic areas studied. These results suggest that sero-epidemiological studies employing MSP-1 (ICB910)-ELISA may be helpful in estimating the prevalence of malaria in certain geographic areas. MSP-1(ICB910)-ELISA can be effectively used to establish and evaluate malaria control and eradication programmes in the affected areas.

## Background

*Plasmodium vivax* causes the relapse of benign tertian human malaria that affects several hundred millions of individuals annually. This disease is a major public health concern in most tropical and many temperate regions, including North and South Korea [1]. The first scientific documentation of malaria occurrence was published in 1913 [2]. A national malaria eradication programme strengthened by the involvement of the World Health Organization has succeeded in significantly reducing the incidence of malaria in South Korea [3, 4]. Malaria was thought to have been eradicated in South Korea in the late 1970s until two sporadic cases were detected in the 1980s [5]. In 1993, a case was diagnosed among South Korean soldiers serving in Northern Gyeonggi Province [6]. Subsequently, Cho *et al.* reported two instances of infected civilians [7]. Thereafter, many new cases have been reported near the demilitarized zone (DMZ): in Paju, Yeoncheon, Cheorwon, Gimpo, Ganghwa, Goyang, and Dongducheon. The increasing number of new cases raises the concern that malaria will become re-established in the region [8, 9].

The malaria research team of the Korea National Institute of Health (KNIH) has developed a new diagnostic method to support pathological examinations. This antibody-based detection method uses merozoite surface protein-1 (MSP-1), an antigen and a large (180–230 kDa) glycoprotein that is synthesized as a precursor to MSP during schizogony [10]. Comparisons of the sequences of MSP-1 from *Plasmodium vivax*, *Plasmodium falciparum* and *Plasmodium yoelii* revealed the existence of ten interspecies conserved blocks (ICBs) containing eight polymorphic regions [11]. Serological surveys have provided valuable epidemiological information, particularly in the areas of low endemicity [12]. Estimation of the rate of parasitaemia is the classical method of measuring the prevalence of malaria. However, the incidence of parasitaemia alone may fail to adequately describe the epidemiology of malaria within a given population. For instance, when the incidence of malaria is low, mass blood surveys do not yield results commensurate with the work involved [13, 14]. In this study, the anti-*P. vivax* MSP-1 antibody levels (particularly against the CB9 to ICB10 region) among the populations of Gimpo, Paju, Yeoncheon, and Cheorwon were determined to evaluate the usefulness of the recombinant MSP-1(ICB910) antigen for assessing the local malaria prevalence.

## Methods

### Blood samples of inhabitants

**Figure 1:**
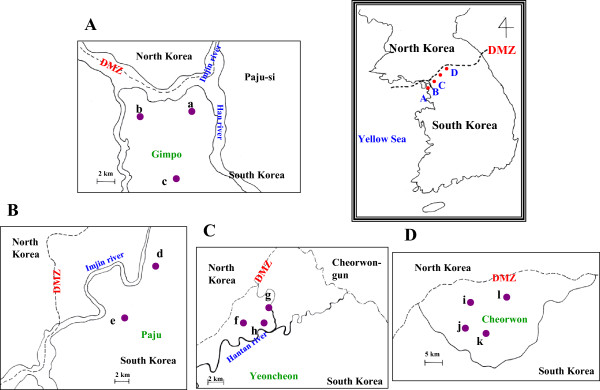
**Blood sample collection areas according to administrative districts. (A)** Gimpo, **(B)** Paju, **(C)** Yeoncheon, **(D)** Cheorwon. **a**, Haseongmyeon; **b**, Wolgotmyeon; **c**, Yangchonmyeon; **d**, Papyeongmyeon; **e**, Munsaneup; **f**, Baekhakmyeon; **g**, Wangjingmyeon; **h**, Misanmyeon; **i**, Gimhwaeup; **j**, Seomyeon; **k**, Cheorwoneup; **l**, Geunnammyeon.

To evaluate the usefulness of the recombinant MSP-1(ICB910) protein in serodiagnosis, blood samples were obtained from the KNIH of the Korean Center for Disease Control and Prevention (KCDC). These blood samples (from 1,774 individuals) were collected from Gimpo and Paju cities, Yeoncheon county of Gyeonggi Province, and Cheorwon of Gangwon Province of South Korea, from November to December of 2004 (Figure 1), and were stored at the KNIH. Blood smears were also obtained from the KNIH for microscopic examination.

### Ethics statement

This study was conducted after receiving the written informed consent from all participants and only after receiving approval from the KNIH. The study procedures, potential risks and benefits were explained to all of them. Further, all data were analysed anonymously and patients were not identified by name. This study was conducted strictly adhering to the principles expressed in the Declaration of Helsinki.

### Microscopic examination

Thin blood films were prepared to determine the infectivity of blood samples. The blood films were fixed with methanol and stained with Giemsa stain to reveal the parasite inclusions in the red blood cells (RBCs). Thin blood films are often preferred for routine estimation of parasitaemia because the organisms can be readily visualized and quantified with this method [15]. To estimate the densities of blood-stage parasites by microscopy, the number of asexual parasites observed per 200 white blood cells (WBCs) was determined, which was then multiplied by the assumed number of WBCs per microlitre of blood (8,000) [16].

### Amplification of the MSP-1 gene

**Figure 2:**
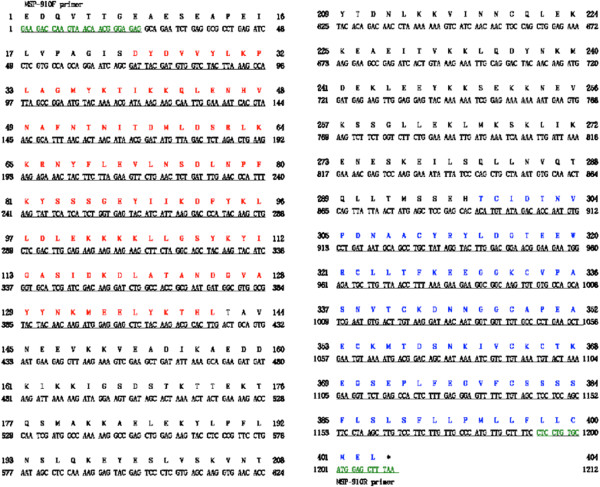
**Nucleotide and amino acid sequences of** ***Plasmodium vivax*** **merozoite surface protein-1(ICB910).** The nucleotide sequence was deposited in GenBank BLAST (http://WWW.ncbi.nlm.nih.gov/nuccore, Accession No. KJ513462).

To express the CB9 to ICB10 region of the *P. vivax* MSP-1 gene, genomic DNA was extracted from the whole blood of a patient diagnosed with malaria using a QIAamp Blood Kit (Qiagen, Hilden, Germany). The polymerase chain reaction (PCR) mixture contained AccuPower PCR PreMix (Bioneer, Daejeon, Korea), 50 ng of purified genomic DNA, and 40 pmoles each of forward (MSP-910 F; 5′-ggatccGAAGACCAAGTAACAACGGGAGAG-3′) and reverse (MSP-910R; 5′-aagcttTTAAAGCTCCATGCACAGGAG-3′) primer (Figure 2). The total volume of the reaction mixture was adjusted to 50 μL with distilled water. The thermal cycling conditions were as follows: denaturation at 94°C for 5 min; 35 cycles of 30 sec at 94°C, 60 sec at 55°C, and 45 sec at 72°C; and a final incubation at 72°C for 5 min. All PCR products were analysed by agarose gel electrophoresis on a 1% agarose gel, visualized under an ultraviolet transilluminator, and purified using a NucleoSpin Extract Kit (Macherey-Nagel; Duren, Germany).

### DNA sequencing and analysis

To genotype the MSP-1(ICB910) gene of *P. vivax*, the MSP-1(ICB910) gene was PCR-amplified and ligated into a pCR2.1 vector (Invitrogen, Carlsbad, CA, USA) and transformed into *Escherichia coli* TOP10 cells. The *E. coli* TOP10 cells containing the recombinant plasmid were selected in medium containing ampicillin [17]. Plasmids were purified using a Qiagen plasmid isolation kit according to the manufacturer’s protocols. The transformants were confirmed by agarose gel electrophoresis following restriction digestion with *Eco*RI. The MSP-1(ICB910) gene sequence was determined using an ABI PRISM Dye Terminator Cycle Sequencing Ready Reaction Kit FS (Perkin Elmer, Cambridge, MA, USA) following manufacturer’s instructions. M13 reverse and forward (-20) primers were used for sequencing. Nucleotide and deduced amino acid sequences were analysed using EditSeq and Clustal in the MegAlign program, a multiple alignment program within the DNASTAR package (DNASTAR, Madison, WI, USA). The internet-based BLAST search program of the National Center for Biotechnology Information was used to search protein databases.

### Construction of the MSP-1(ICB910) expression vector

To express the MSP-1 gene in *E. coli* DH5α cells, the CB9 to ICB10 region of the MSP-1 gene fragment was amplified using MSP-910 F and MSP-910R primers (respectively containing *Bam*HI and *Hin*dIII sites at their 5′ ends) from a blood sample that was confirmed to be infected with the dormant type of *P. vivax* (Figure 2). The amplified-PCR products were digested with *Bam*HI and *Hin*dIII, the products were gel-purified using a Qiagen gel extraction kit, and were ligated between the *Bam*HI and *Hin*dIII cleavage sites of the pQE30 expression vector (Qiagen). The resulting plasmid was used for the expression of a MSP-1(ICB910)-(His)_6_ fusion protein in *E. coli* cells. The transformants were first confirmed by agarose gel electrophoresis following restriction digestion using *Bam*HI and *Hin*dIII. Finally, the insert sequence was confirmed by DNA sequencing.

### Expression and purification of recombinant MSP-1(ICB910)

The expression of the recombinant MSP-1(ICB910)-(His)_6_ fusion protein in *E. coli* DH5α cells was induced with isopropyl-1-thio-β-D-galactopyranoside (IPTG) [18]. The MSP-1(ICB910)-(His)_6_ fusion protein was purified using immobilized metal ion affinity chromatography [19] under native conditions following the manufacturer’s protocols (Qiagen). Protein levels were analysed by sodium dodecyl sulphate-polyacrylamide gel electrophoresis (SDS-PAGE) after each purification step.

### Western blot analysis

The recombinant MSP-1(ICB910)-(His)_6_ fusion protein was separated by SDS-PAGE on a 12% gel and transferred onto a nitrocellulose membrane. After the transfer, the membrane was cut into strips and blocked for nonspecific binding with 3% skim milk for 12 hr at 4°C. The membrane was then washed three times for 10 min each with 0.15% Tween 20 in phosphate-buffered saline (PBS). Following this, the strips were allowed to react for 4 hr with sera from patients with malaria or from uninfected individuals (diluted 1:100, vol/vol). The membranes were then washed three times for 10 min each with 0.15% Tween 20 in PBS and were subsequently incubated with peroxidase-conjugated goat anti-human IgG secondary antibody (1:1,000) (Sigma) for 3 hr at room temperature. For colour development, a solution containing 0.2% diaminobenzidine and 0.02% H_2_O_2_/PBS was applied to each well [20, 21].

### Enzyme-linked immunosorbent assay (ELISA)

An ELISA was used to determine whether the blood samples contained antibodies against MSP-1(ICB910) antigens. Briefly, the capture antigen solution (50 μL, 0.5 μg/mL) was placed in a 96-well plate (Corning, Lowell, MA, USA) and incubated for 12 hr at room temperature. The antigen solution was then aspirated, blocking buffer (1% bovine serum albumin, 0.05% PBS-Tween 20) was added to each well, and the plate was incubated for 1 hr at room temperature. After the wells were washed three times with 0.05% Tween 20/PBS, human serum samples in blocking buffer at a dilution of 1:100 (vol/vol) were added to the wells. Four positive and four negative control serum samples were also included in each plate. After incubating at room temperature for 2 hr, the plates were washed with 0.05% Tween 20/PBS three times. Peroxidase-conjugated anti-human IgG (Sigma, 1:2,000, vol/vol) diluted in blocking buffer was then added to each well and the plates were incubated for 1 hr at room temperature. Following this, the reaction was stopped by washing the plates as described above. To develop the colour, 100 mL of 2,2′-azino-di-(3-ethyl-benzthiozoline-6-sulphonic acid) (ABTS) peroxidase substrate (Kirkegaard & Perry Laboratories, Gaithersburg, MD, USA) was added to each well and the plates were incubated for 30 min. This was followed by measuring the absorbance of the solution at 405 nm. Cut-off value for ELISA-positivity was defined as the sum total of the mean values and two times the standard deviations of the negative control samples.

### Estimation of the annual parasite incidence (API)

For each of the study sites, the annual parasite incidence (API) was calculated as the number of malaria-positive patients per 1,000 inhabitants; API = (number of positive slides/total number of slides) × 1,000.

### Data analysis

The data were analysed using Graphpad Prism software version 4.0 (GraphPad Software Inc., La Jolla, CA, USA). Pearson’s correlation analysis was performed to examine the relationship between seropositivity and the API of *P. vivax* in a given year. The data were analysed using SPSS software, version 17.0 (SPSS Inc., Chicago, IL, USA). A *P-*value of <0.05 indicated statistical significance. The correlation sizes were interpreted as none (0.0-0.09), small (0.1-0.3), medium (0.3-0.5), or strong (0.5-1.0) [22].

## Results

### Blood sampling

The study locations are shown on the map in Figure 1. All areas were near the DMZ, a known high-risk area. Blood samples were collected from participants residing in 23 villages and three cities (Gimpo, Paju and Yeoncheon) located in Gyeonggi Province and six villages in Cheorwon of Gangwon Province, South Korea. A total of 1,774 blood samples (1.92%) were collected. The total number of inhabitants in the geographical areas where the study was conducted in the year 2004 was 92,246.

### DNA sequence of *Plasmodium vivax*MSP-1(ICB910) (Korean isolate)

The region from CB9 to ICB10 of the MSP-1 gene that was PCR-amplified from the genomic DNA was analysed on a 1.0% agarose gel. Amplification of the MSP-1(ICB910) gene yielded an approximately 1,200-bp DNA fragment that was then ligated to the pCR2.1 cloning vector. Restriction analysis using EcoRI confirmed the identity of the transformants. The plasmid containing the PCR product was named pMSP910 and was subjected to DNA sequence analysis. DNA sequencing revealed that the cloned MSP-1(ICB910) gene was 1,212-bp in length and encoded 403 amino acids as identified by DNASIS (Figure 2, Genbank accession No KJ513462).

### Expression of MSP-1(ICB910) in *Escherichia coli*and its antigenicity

**Figure 3:**
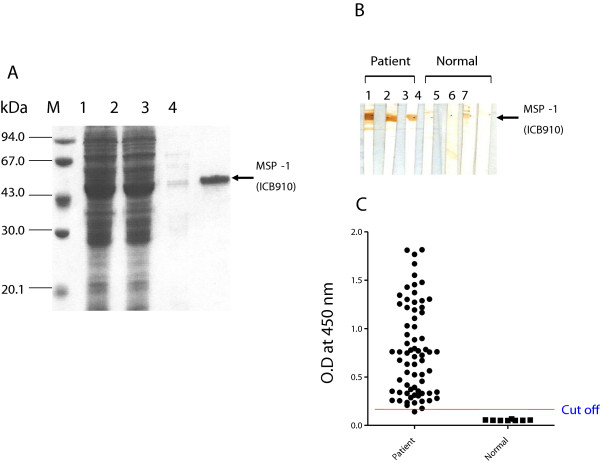
**Characterization of recombinant merozoite surface protein-1 (ICB910). (A)** Purification of recombinant MSP-1 (ICB910) by Ni-NTA agarose affinity chromatography. Lane M, molecular weight marker; lane 1, *E. coli* DH5α cell lysate after protein expression was induced with IPTG; lane 2, flow-through; lane 3, wash; lane 4, eluted fraction. **(B)** Western blot analysis of recombinant MSP-1(ICB910) protein. Lanes 1–3, samples from patients infected with malaria; Lanes 4–7, samples from uninfected individuals. **(C)** Immune responses of recombinant MSP-1(ICB910) to vivax malaria patient group and normal person by Enzyme linked immunosorbent assay.

To construct the expression plasmid, the MSP-1(ICB910) gene was amplified from the patient’s genomic DNA, digested with *Bam*HI and *Hin*dIII, and subcloned into the same restriction enzyme sites of the pQE30 expression vector to produce pMSPex910 containing a (His)_6_-tag. The recombinant pMSPex910 plasmid was then transferred into *E. coli* DH5α cells. For protein expression, 1 mM IPTG was added to *E. coli* DH5α (pMSPex910) cells grown to logarithmic phase in liquid Luria-Bertani medium containing 100 μg/mL ampicillin and 50 μg/mL kanamycin to induce the expression. SDS-PAGE followed by Coomassie blue staining showed that under native purification conditions, the molecular weight of the recombinant MSP-1(ICB910) protein was ~46 kDa (Figure 3A). The antigenicity of the MSP-1(ICB910) recombinant protein was determined by Western blot. The sera from patients with malaria reacted positively (Figure 3B, No 1–3). One sample (Figure 3B, No 6), which was collected from an individual who had no symptoms of infection, showed weak positivity. During the follow-up study, this case became ill four months after the blood sampling and was diagnosed with vivax infection. To determine the sensitivity and specificity of the MSP-1(ICB910) recombinant protein by ELISA, the sera of patients with malaria were used. Sera from 68 of the 70 patients with malaria (sensitivity, 97.1%) were ELISA-positive, whereas all samples in the normal control group (n = 8) were ELISA-negative (specificity, 100%) (Figure 3C).

### Overview of malaria transmission in four locations

One-hundred and forty-six of the 1,774 study subjects (8.23%) showed MSP-1(ICB910)-ELISA positivity. Yeoncheon presented the highest positive rate (Figure 1C, 46/451, 10.20%), followed by Paju (Figure 1B, 35/372, 9.41%), Cheorwon (Figure 1D, 44/526, 8.37%), and Gimpo (Figure 1A, 21/425, 4.94%). The 2004 API was higher than the 2005 API. API did not vary within the same geographic location during the years 2004 and 2005. Yeoncheon presented the highest API in both years, followed by Cheorwon, Paju and Gimpo. The seropositivity for the year 2004 showed a strong positive linear relationship with the APIs of 2004 and 2005 (r = 0.691 and r = 0.842, respectively), but was statistically insignificant (Table 1).

**Table 1:** **Rates of MSP-1(ICB910)-ELISA positivity and annual parasite incidence**

Area	No. of sera tested	No. of positive sera	Positive rate (%)	API ^a^	
				2004	2005
Gimpo	425	21	4.94	0.73	0.28
Paju	372	35	9.41	1.07	0.86
Yeoncheon	451	46	10.20	2.99	1.99
Cheorwon	526	44	8.37	1.13	1.20
Total	1774	146	8.23	1.48	1.08

^a^ *API*; Annual parasite incidence.

-Correlation coefficient between MSP-1(ICB910) positive rate of 2004 and API of 2004 (r = 0.691, *P =* 0.309).

-Correlation coefficient between MSP-1(ICB910) positive rate of 2004 and API of 2005 (r = 0.842, *P =* 0.158).

### Local malaria transmission in Gimpo

Twenty-one of the 425 inhabitants (4.94%) presented a positive response in MSP-1(ICB910)-ELISA. Haseongmyeon presented the highest ELISA-positive rate (Figure 1a, 6/53, 11.32%), followed by Yangchonmyeon (Figure 1c, 10/206, 4.85%) and Wolgotmyeon (Figure 1b, 5/166, 3.01%). Wolgotmyeon presented the highest API in 2004 (1.42) and 2005 (0.71). Haseongmyeon presented the second highest API in 2004 (0.69), but there were no new diagnoses in 2005. Yangchonmyeon was third in API in 2004 (0.50) and second in the year 2005 (0.25). The seropositivity in 2004 showed a strong negative linear relationship with the APIs of the years 2004 and 2005 (r = -0.513, r = -0.887, respectively). However, these correlations were not statistically significant (Table 2).

**Table 2:** **Rates of MSP-1(ICB910)-ELISA positivity and annual parasite incidence in Gimpo**

Village	No. of sera tested	No. of positive sera	Positive rate (%)	API ^a^	
				2004	2005
Haseongmyeon	53	6	11.32	0.69	0.00
Yangchonmyeon	206	10	4.85	0.50	0.25
Wolgotmyeon	166	5	3.01	1.42	0.71
Total	425	21	4.94	0.87	0.49

^a^ *API*; Annual parasite incidence.

-Correlation coefficient between MSP-1 (ICB910) positive rate of 2004 and API of 2004 (r = -0.513, *P =* 0.657).

-Correlation coefficient between MSP-1 (ICB910) positive rate of 2004 and API of 2005 (r = -0.887, *P =* 0.306).

### Local malaria transmission in Paju

Thirty-five of the 372 inhabitants (9.41%) showed an ELISA-positive response. Musaneup had a higher positive rate (Figure 1e, 28/232, 12.07%) than Papyeongmyeon (Figure 1d, 7/140, 5.00%). Papyeongmyeon had a higher API than Munsaneup in the years 2004 and 2005. The seropositivity in 2004 showed a strong negative linear relationship with the APIs of 2004 and 2005 (r = -1.000 and r = -1.000, respectively). This result was statistically significant (*P* = 0.01 for each year, Table 3).

**Table 3:** **Rates of MSP-1(ICB910)-ELISA positivity and annual parasite incidence in Paju**

Village	No. of sera tested	No. of positive sera	Positive rate (%)	API ^a^	
				2004	2005
Munsaneup	232	28	12.07	1.00	0.82
Papyeongmyeon	140	7	5.00	1.60	1.20
Total	372	35	9.41	1.31	1.01

^a^ *API*; Annual parasite incidence.

-Correlation coefficient between MSP-1(ICB910) positive rate of 2004 and API of 2004 (r = -1.000, *P =* 0.01).

-Correlation coefficient between MSP-1(ICB910) positive rate of 2004 and API of 2005 (r = -1.000, *P =* 0.01).

### Local malaria transmission in Yeoncheon-gun

Forty-six of the 451 inhabitants (10.20%) of Yeoncheon-gun showed an ELISA-positive response. Misanmyeon had the highest positive rate (Figure 1h, 9/75, 12.00%), followed by Baekhakmyeon (Figure 1f, 28/265, 10.57%) and Wangjingmyeon (Figure 1g, 9/111, 8.11%). Baekhakmyeon had the highest API in 2004 (3.69) and dropped to third place in 2005 (1.34). Misanmyeon had the second highest API in 2004 (2.79) and ranked first in 2005 (2.79). Wangjingmyeon had the lowest API in 2004 (1.60) and ranked second in 2005 (2.40). The seropositivity in 2004 showed a strong positive linear relationship with the API of 2004 (r = 0.685) and a weak linear relationship with API of 2005 (r = 0.111). However, these results were statistically insignificant (Table 4).

**Table 4:** **Rates of MSP-1(ICB910)-ELISA positivity and annual parasite incidence in Yeoncheon**

Village	No. of sera tested	No. of positive sera	Positive rate (%)	API ^a^	
				2004	2005
Wangjingmyeon	111	9	8.11	1.60	2.40
Baekhakmyeon	265	28	10.57	3.69	1.34
Misanmyeon	75	9	12.00	2.79	2.79
Total	451	46	10.20	2.69	2.18

^a^ *API*; Annual parasite incidence.

-Correlation coefficient between MSP-1(ICB910) positive rate of 2004 and API of 2004 (r = 0.685, *P =* 0.519).

-Correlation coefficient between MSP-1(ICB910) positive rate of 2004 and API of 2005 (r = 0.111, *P =* 0.929).

### Local malaria transmission in Cheorwon

Forty-four of the 526 inhabitants (8.37%) of Cheorwon showed MSP-1(ICB910)-ELISA positivity. Cheorwoneup presented the highest positive rate (Figure 1k, 17/142, 11.97%), followed by Geunnammyeon (Figure 1l, 15/143, 10.49%), Gimhwaeup (Figure 1i, 12/115, 10.43%) and Seomyeon (Figure 1j, 0/126, 0.00%). Seomyeon had the highest API in 2004 (1.98), followed by Gimhwaeup (1.54), Cheorwoneup (0.86) and Geunnammyeon (0.45). However, Gimhwaeup had the highest API in 2005, followed by Geunnammyeon (2.23), Seomyeon (0.49) and Cheorwoneup (0.35). The seropositivity in 2004 showed a strong negative linear relationship with an API of 2004 (r = -0.762) and a strong positive linear relationship with API of 2005 (r = 0.430). However, these correlations were statistically insignificant (Table 5).

**Table 5:** **Rates of MSP-1(ICB910)-ELISA positivity and annual parasite incidence in Cheorwon**

Village	No. of sera tested	No. of positive sera	Positive rate (%)	API ^a^	
				2004	2005
Gimhwaeup	115	12	10.43	1.54	2.46
Seomyeon	126	0	0.00	7.98	0.49
Geunnammyeon	143	15	10.49	0.45	2.23
Cheorwoneup	142	17	11.97	0.86	0.35
Total	526	44	8.37	1.21	1.38

^a^ *API*; Annual parasite incidence.

-Correlation coefficient between MSP-1(ICB910) positive rate of 2004 and API of 2004 (r = -0.762, *P =* 0.238).

-Correlation coefficient between MSP-1(ICB910) positive rate of 2004 and API of 2005 (r = 0.420, *P =* 0.580).

## Discussion

The areas surveyed, Gimpo, Paju, Yeoncheon, and Cheorwon, which are located within 10–15 km of the southern DMZ, are part of re-emerging malarial outbreak areas in South Korea [8]. The DMZ is a 4 km-wide and 250 km-long corridor that extends across the middle part of the Korean peninsula. Natural landscape, ecosystems and biodiversity are highly conserved in the DMZ [23]. The outbreak areas expanded yearly both in the southern and eastern directions from the DMZ in the initial stage of re-emergence. Although there has been a sharp decline in the reported cases in recent years, a re-emergence of malaria in these areas cannot be ruled out. Effective control programmes will prevent the re-emergence of malaria. Sensitive diagnostic tools that allow rapid and accurate diagnosis will ensure the effectiveness of malaria prevention and control programmes. MSP-1 is considered as a useful antigen for serodiagnosis and is an important vaccine candidate against asexual blood stages [24–27]. The MSP-1 protein binds to the surface of erythrocytes and is one of the merozoite surface ligands involved in the invasion of the erythrocytes. Comparison of the sequences of MSP-1 proteins from *P. vivax*, *P. falciparum*, and *P. yoelii* revealed seven ICBs (ICB1, ICB2, ICB4, ICB5, ICB6, ICB8, and ICB10) and three CBs (CB3, CB7 and CB9) [11]. The immune responses to the N- and C-terminal regions of MSP-1 have been characterized [24]. The 11 regions of MSP-1 expressed in *E. coli* were glutathione S-transferase (GST) regions. It was reported that 83.3% of malaria patients had IgG complexes against at least one of the GST-fusion proteins. In addition, the frequency of patients with the IgG antibodies to recombinant ICB10 protein, which contained only the 111 C-terminal amino acids of MSP-1, increased with the number of *P. vivax* malaria experiences, reaching 83.3% after four experiences. However, the responses to recombinant ICB2-5, which consisted of 506 C-terminal amino acids of MSP-1, did not have the same frequency. The titre of the antibody against recombinant ICB10 protein was greater than that of the antibody against recombinant ICB2-5. Furthermore, ICB10 helps peripheral blood mononuclear cells (PBMCs) to secrete IFN-γ, suggesting that T-cell epitopes are present in this region. Soares *et al.* found that the C-terminal region was immunogenic to both antibodies and T-cells were produced following infections in humans [28]. MSP-1 could be a useful vaccine against *P. vivax* malaria because the C-terminal region of MSP-1 is less polymorphic than the N-terminal region [29] and the epitopes to B- and T-cells have specific humoral responses that produce longer-term stability [30]. Therefore, the region from CB9 to ICB10 of MSP-1 was selected and expressed as a recombinant protein in *E. coli* for use in sero-epidemiology. In the present study, a recombinant MSP-1(ICB910) antigen-based ELISA diagnostic method was used to evaluate the antibody levels in the inhabitants of high-risk areas. The incidence of malaria peaks in August after the rainy season and declines to baseline by mid-October. Therefore, blood sampling was carried out between late-October and mid-December, when the active anopheline population had diminished. Since ELISA-based screening for the presence of an antibody could provide useful information regarding *P. vivax* infection in a previously naïve population, the significance between the positive rate and incidence of malaria in high-risk areas were compared.

The MSP-1(ICB910)-ELISA-positive rates and the APIs of four cities in both years showed strong linear relationships, but were not statistically significant (*P* = 0.309 and *P* = 0.158, respectively) (Table 1). The local transmission in Gimpo showed a strong negative relationship between API and MSP-1(ICB910)-ELISA, but it was not significant (2004, *P* = 0.657; 2005, *P* = 0.306). Local transmission in Paju showed a strong negative relationship between API and MSP-1(ICB910)-ELISA that was statistically significant (2004, *P* = 0.01; 2005, *P* = 0.01). Local transmission in Yeoncheon showed strong relationship between the API and MSP-1(ICB910)-ELISA of 2004 and a weak relationship in 2005 (2004, *P* = 0.519; 2005, *P* = 0.929). In Cheorwon, the MSP-1(ICB910)-ELISA response and API had a strong negative relationship in 2004 (*P* = 0.238) and a medium positive relationship in 2005 (*P* = 0.580). These differences might be related to the different malaria control programmes adopted in the areas. Malaria control programmes are administered by four public health centres (PHCs). Each PHC uses their own manual that takes the local malaria prevalence and geographical characteristics into account. While evaluating the malaria transmission in a given geographical region, factors such as temperature, mosquito density, vector capacity, climate, rainfall, and humidity must be considered [31]. However, collecting data requires much effort. Therefore, it is necessary to establish a cost-effective tool to analyse the current and future transmission of malaria in specific geographic areas using serodiagnostic methods. Parasitaemia provides a classical means for measuring malaria endemicity, but patient incidence alone is unlikely to provide a complete understanding of malaria prevalence. Factors including the population density of mosquitoes, vectorial capacity, long- to short-incubation patient ratio, symptomatic to asymptomatic patient ratio, differences in rainfall and temperature, and immunity of the community all affect malaria prevalence in Korea. These results suggest that antibody detection methods provide results that are more valuable than API values obtained by microscopic examination in certain circumstances. Such methods will help to better understand malaria transmission in a given geographical area.

## Conclusions

The antibody detection method using MSP-1 (ICB910)-ELISA provides useful information regarding malaria prevalence in specific geographical areas and individuals. This serological method is useful for identifying areas that require malaria control.
